# Drivers of seedling survival in a temperate forest and their relative importance at three stages of succession

**DOI:** 10.1002/ece3.1688

**Published:** 2015-09-10

**Authors:** Yan Yan, Chunyu Zhang, Yuxi Wang, Xiuhai Zhao, Klaus von Gadow

**Affiliations:** ^1^ Key Laboratory for Forest Resources & Ecosystem Processes of BeijingBeijing Forestry UniversityBeijing100083China; ^2^ Division of Forestry and Natural ResourceWest Virginia UniversityMorgantownWest Virginia; ^3^ Georg‐August UniversityGöttingenGermany; ^4^ Department of Forest and Wood ScienceUniversity of StellenboschStellenboschSouth Africa

**Keywords:** Community compensatory trend, negative density dependence, niche partitioning, seedling survival

## Abstract

Negative density dependence (NDD) and niche partitioning have been perceived as important mechanisms for the maintenance of species diversity. However, little is known about their relative contributions to seedling survival. We examined the effects of biotic and abiotic neighborhoods and the variations of biotic neighborhoods among species using survival data for 7503 seedlings belonging to 22 woody species over a period of 2 years in three different forest types, a half‐mature forest (HF), a mature forest (MF), and an old‐growth forest (OGF), each of these representing a specific successional stage in a temperate forest ecosystem in northeastern China. We found a convincing evidence for the existence of NDD in temperate forest ecosystems. The biotic and abiotic variables affecting seedlings survival change with successional stage, seedling size, and age. The strength of NDD for the smaller (<20 cm in height) and younger seedlings (1–2 years) as well as all seedlings combined varies significantly among species. We found no evidence that a community compensatory trend (CCT) existed in our study area. The results of this study demonstrate that the relative importance of NDD and habitat niche partitioning in driving seedling survival varies with seedling size and age and that the biotic and abiotic factors affecting seedlings survival change with successional stage.

## Introduction

Understanding the ecological processes that drive community assembly and their relative contributions remains a major challenge for ecologists (Paine et al. 2012; Comita et al. 2014). A number of theories have sought to explain the mechanisms of species coexistence. Resource‐based niche partitioning and negative density dependence are two of the widely discussed mechanisms contributing to the maintenance of diversity (Hutchinson 1961; Janzen 1970; Connell 1978). For tree communities, the transition from seedling to sapling has been seen as a bottleneck in tree establishment (Queenborough et al. 2007). Compared with mature plants, juveniles suffer most from both biotic and abiotic constraints (Wright 2002) and the relative importance of these mechanisms is expected to change over time (Comita et al. 2009). As a consequence, much attention has been given to the seedling stage.

Under the assumption of the Janzen–Connell hypothesis, host‐specific natural enemies, that is, seed predators, pathogens, and herbivores reduce survival and recruitment of seedlings when they occur in localized, conspecific density (LCD) areas (Janzen 1970; Connell et al. 1971). In addition, an asymmetric resource competition with conspecific nearby adults may result in high seedling mortality. At the community level, local‐scale NDD manifests itself as a community compensatory trend (CCT, Connell et al. 1984) where common species will be more likely to have a greater chance of encountering conspecifics among their neighborhoods and may undergo stronger NDD compared to less abundant species (Metz et al. 2010). As a result, the more common species are assumed to have higher rates of mortality than the rare species, resulting in an advantage for the rare species. However, Comita et al. (2010) could show that the strength of local‐scale NDD may vary among species and decreases with increasing species abundance. In consequence, rare species suffered a higher mortality than common species when they had similar LCD. Therefore, the variation among species in the strength of NDD is a crucial factor that should be taken into consideration when assessing the role of local‐scale NDD in shaping species abundances (Lin et al. 2012).

Recent studies of seedling survival as a function of local biotic neighborhoods have found widespread NDD and CCT consistent with the Janzen–Connell hypothesis in both subtropical and tropical tree communities (Harms et al. 2000; Volkov et al. 2005; Bell et al. 2006; Zhu et al. 2010; Comita et al. 2014; Jansen et al. 2014). These studies point to NDD as an important stabilizing force promoting species coexistence in forest ecosystems. Furthermore, it has been demonstrated that NDD is an important mechanism in temperate forests for maintaining community diversity (Packer and Clay 2000; Hille Ris Lambers et al. 2002).

Habitat niche partitioning is another factor that impacts seedling survival (Webb and Peart 2000). According to the niche theory, functional differences among species may be a result of evolutionary adaptation to interspecific competition for limited environmental resources (Gillespie 2004; Harpole and Tilman 2006). These interspecific trade‐offs promote differences in resource requirements among species and affect the competitive ability of species across heterogeneous environments (Schoener 1974 Vergnon et al. 2009). As species can be differentiated by particular combinations of a local abiotic environment, such as light, soil, water, and soil nutrients (Silvertown 2004; Adler et al. 2007), the niche process would exhibit a positive interaction between species adaptability and population size when growing in its preferred habitat (Wright 2002). In this situation, if host‐specific natural enemies or intraspecific competition cannot offset their habitat advantages, a positive relationship between LCD and seedling survival would be found, despite an underlying NDD (Piao et al. 2013).

A large number of theories attempts to explain the mechanisms of species coexistence, with empirical and experimental support (Harms et al. 2001; Russo et al. 2005; Lutz et al. 2014; Jansen et al. 2014), and researchers are convinced that the theories are not all mutually exclusive (Hubbell 2005; Gravel et al. 2006; Queenborough et al. 2009). Existing evidence is still insufficient to settle some of the conflicts between the different theories of species coexistence (Martorell et al. 2014) which have rarely been investigated concurrently (Bin et al. 2011; Chanthorn et al. 2013), especially in temperate forests. Moreover, these mechanisms may change throughout the life of an organism. Different factors become dominant at various stages and have diverse effects. Previous studies examining the mechanisms that drive seedling survival patterns did focus on natural enemies and resource limitation, regardless of their sensitivity to those factors. The larger individuals have lower mortality rates (Winkler et al. 2005; Ratikainen et al. 2008), because larger individuals may be more resistant and resilient to biotic and abiotic stresses (Bai et al. 2012). Therefore, to determine the relative importance of density dependence and niche processes that may be responsible for seedling survival in a relatively diverse temperate tree community in northeastern China, seedling size must be considered.

In this study, we examine the effects of biotic and abiotic neighborhoods and the variation in the effect of biotic neighborhoods among species for seedling survival in a temperate community in northeastern China. We evaluate their relative importance over different successional stages, size classes, and ages, using observations on a total of 7503 seedlings of 22 woody species in three different of forest successional stages. In particular, we are looking for answers to the following questions: (1) Does the effect of biotic and abiotic neighborhoods on seedling survival differ among seedling size classes (seedling <20 cm in height or seedling ≥20 cm in height), ages, and successional stages? (2) At a local scale, is seedling survival negatively affected by an increasing, conspecific neighborhood density and is this manifested as CCT at the community level? (3) Does the strength of NDD vary among species? (4) Which of the ecological processes, NDD or habitat niche partitioning determines the pattern of tree seedling survival?

## Materials and Methods

### Study area

The study was carried out in a typical temperate mixed broadleaf–conifer forest in Jilin province, northeastern China, in an experimental forest, located at (43°51′–44°05′N, 127°35′–127°51′E) which is under the jurisdiction of the Jiaohe Administrative Bureau (Zhao et al. 2014, Fig. 1). The climate is characterized by a continental influence during the winter months originating from the interior of the Asian continent and a temperate monsoon climate in summer originating from the western Pacific. The mean annual temperature is 3.8°C, with the mean daily temperature ranging from −18.6°C in January to 21.7°C in July. The mean annual precipitation is 695.9 mm. The dominant soil type is a brown forest soil.

**Figure 1:**
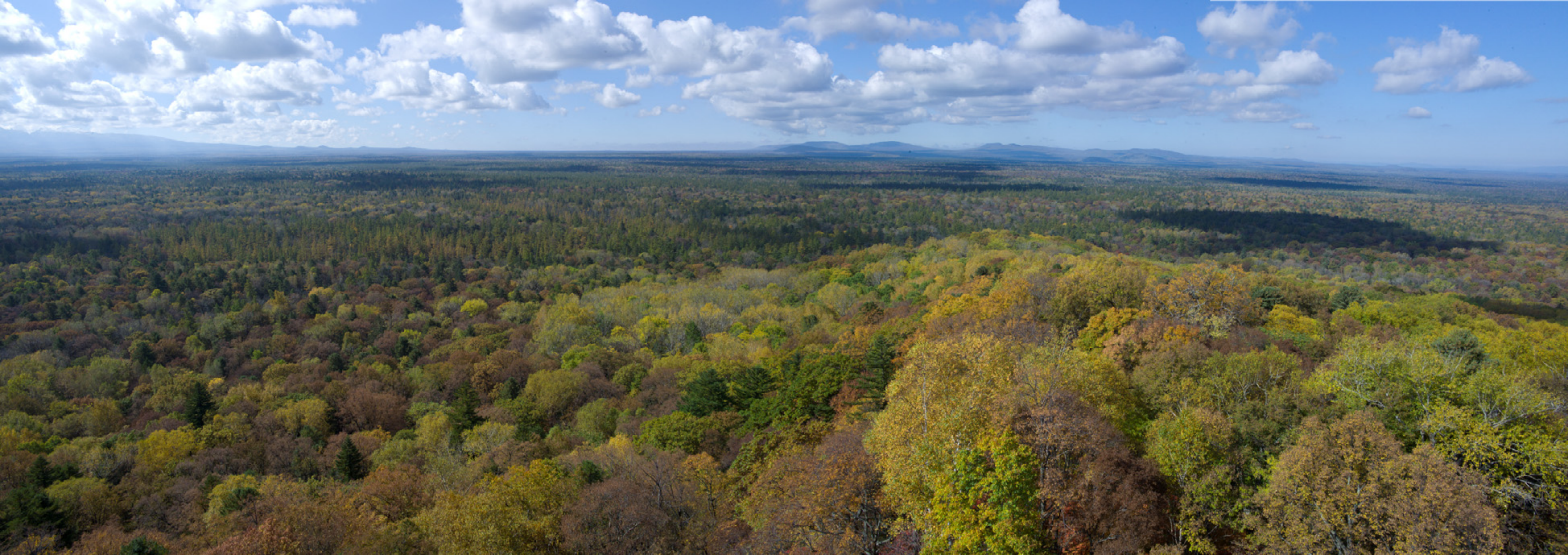
An aerial view of broad leaved *Pinus Koraiensis* mixed forest in Jiaohe, Jilin province, northeastern China (Photographed by ZHAO Xiu‐Hai). The broad leaved *Pinus Koraiensis* mixed forest is the typical zonal vegetation type in the northeastern China, which is in the climax stage of community succession. The major tree species include *Pinus koraiensis*,* Acer mono*,* Tilia amurensis*,* Juglans mandshurica,* and *Fraxinus mandshurica*.

This study is based on observations collected in three large permanent field plots established in 2010. The observational studies belonging to three successional stages: a half‐mature forest (HF, 21.84 ha), a mature forest (MF, 42 ha), and an old‐growth forest (OGF, 30 ha). Within each plot, all woody species with a diameter at breast height (DBH) equal or greater than 1 cm had been tagged, mapped, measured, and identified by species in the summer of 2010.

The HF plot is situated in a secondary broadleaf–conifer forest in the primary stages of succession. The forest was clear‐cut about 60 years ago. The topography is flat with elevations ranging from 468 to 519 m above sea level. There is a total of 29,035 living trees and shrubs ≥1 cm DBH belonging to 17 families 26, genera, and 42 species. The top five species in basal area are *Juglans mandshurica* Maxim., *Fraxinus mandshurica* Rupr., *Ulmus davidiana Planch*. var. *japonica* (Rehder) Nakai, *Acer mono* Maxim., and *Pinus koraiensis* Siebold & Zucc. (Table S1).

The MF plot is located in a secondary broadleaf–conifer forest in the middle stage of succession. The forest was heavily disturbed by forest management about 60 years ago, and most canopy trees are now 100–120 years old (Zhang et al. 2014). The topography in the MF is usually flat with elevations ranging from 459 to 517 m above sea level. There are a total of 55,501 living trees and shrubs ≥1 cm DBH belonging to 17 families, 28 genera, and 46 species. The top five species in base area are *A. mono*,* P. koraiensis*,* J. mandshurica*,* F. mandshurica*, and *Tilia amurensis* Rupr. (Table S1).

The OGF plot is situated in a protected old‐growth forest in the late stage of succession, far away from residential areas, with little human disturbance. The topography includes a valley between two slopes with elevations ranging from 576 to 784 m above sea level. The study area includes a total of 49,090 living trees and shrubs ≥1 cm DBH belonging to 18 families, 30 genera, and 47 species. The top five species in base area are *Ulmus laciniata* (Trautv.) Mayr., *A. mono*,* T. amurensis*,* P. koraiensis,* and *Betula costata* Trautv. (Table S1).

### Seedling census

A total of 451 census stations were established during 2011, in a systematic sampling design to monitor seed rain and seedling dynamics in each of the three plots: 99 such stations were set up in the HF, 209 in the MF, and 143 in the OGF study area (Table 1). Each station consists of a 0.64‐m^2^ seed trap for collecting seeds and litter, with four 1‐m^2^ seedling quadrats, established at a distance of 1 m from each of the four sides of each seed trap. Figure S1 shows the layout of a station. The first seedling census took place in July and August 2012, with all woody seedlings <1 cm DBH (referred to simply as “seedlings”) within each quadrat marked, identified, measured for height to the apical bud (cm) and basal stem diameter (mm), and measured at ground level. The age of each seedling was estimated by counting annual bud scale scars. The seedling quadrats were resampled after 1 year to check the survival and growth of previously marked seedlings and to measure newly recruited seedlings.

**Table 1:** Summary for permanent forest plots

Forest type	Plot area	Latitude	Elevation	No. of stations
HF	21.84 ha	43°58.383′ N	468 m – 519 m	99
	(420 m × 520 m)	127°44.317′ E		
MF	42.00 ha	43°57.783′ N	459 m – 517 m	209
	(500 m × 840 m)	127°44.389′ E		
OGF	30.00 ha	43°58.071′ N	576 m – 784 m	143
	(500 m × 600 m)	127°45.539′ E		

### Biotic neighborhood density variables

In 2012, the seedling density in each of the 1804 quadrats (451 stations × 4 quadrats per station) was obtained. The density of adult neighborhoods (A), involving mature trees, was calculated as the sum of the basal areas (BA) of each conspecific (Cona) or heterospecific (Heta) adult within 10 m from the trap center, divided by the distance of each mature tree to the trap center:

$$A=\sum_{i}^{N}{\text{BA}}_{i}/{\text{Distance}}_{i},$$

where *i* is a neighboring mature tree; BA its basal area (cm^2^), and distance is the distance (m) between the trap and the *i* th tree.

### Abiotic neighborhood variables

Canopy openness and soil properties were used as abiotic neighborhood variables at each seedling station. In August 2012, canopy openness was determined from hemispherical canopy photographs at the center of each station, using a Nikon Coolpix 4500 camera body (Nikkor; Nikon Inc., Tokyo, Japan) and a Nikon FC‐E8 Fisheye Converter lens (Nikkor; Nikon Inc.). Photographs were taken 1.5 m above ground level under overcast conditions. Images were analyzed using the programs WinSCANOPY and XLScanopy. The mean canopy openness values were 1.95 ± 0.42 (SD) in the case of HF, 2.13 ± 0.52 (SD) in MF and 2.10 ± 0.66 (SD) in OGF.

We collected soil samples at each station at a depth of 0–10 cm for the analysis of chemical properties. Eight soil nutrients were recorded as follows: pH, the amount of organic matter, and the total amounts as well as the available nutrients of nitrogen (N), phosphorus (P), and potassium (K). All laboratory analyses were conducted following the procedures recommended by the Soil Science Society of China (Soil Science Society of China, 1999). In order to reduce the number of variables describing soil factors, we used principal component analysis (PCA) to identify major trends in soil variables.

In the HF study area, the first five components produced by the PCA explained 86% of the variation in soil conditions (Table S3). The first PCA axis (henceforth referred to as PC1) was positively related with total K and negatively related with organic matter, pH, total N, P, and available N, P, K. The second PCA axis (henceforth referred to as PC2) was positively related with total N and pH and negatively with total K and available N, P, K. The third PCA axis (henceforth referred to as PC3) indicated a positive response to organic matter, total N, P, and available P and a negative one to pH, total K and available N, K. The fourth PCA axis (henceforth referred to as PC4) was positively related with total P, available K and pH and negatively with organic matter, total N, K, and available P. The fifth PCA axis (henceforth referred to as PC5) was positively related with available N, K and negatively with organic matter, pH, total P, K, and available P.

In the MF study area, the first five components produced by the PCA explained 85% of the variation in soil conditions (Table S3). The PC1 was positively related with total K and negatively with organic matter, pH, total N, P, and available N, P, K. The PC2 was positively related with organic matter, pH, total N, K, and available N, P and negatively with total P and available K. The PC3 was positively related with organic matter, total N and negatively with total P and available N, P. The PC4 was positively related with total N, P, K and negatively with available K and pH. The PC5 was positively related with total K, P and available P and negatively related with total N and available P.

In the OGF study area, the first five components produced by the PCA explained 80% of the variation in soil conditions (Table S3). The PC1 was positively related with organic matter, pH, total N and available N, P. The PC2 was positively related with total N and negatively with total P, available P, K, and pH. The PC3 was positively related with available N, P and total N and negatively with pH, total N, K and available K. The PC4 was positively related with available K and pH and negatively related with total P, K and available N, P. The PC5 was positively related with available N, P, K and negatively with total P and pH.

### Statistical analysis

To assess the relative importance of different processes on seedling survival, we simulated the probability of individual seedling survival as a function of neighborhood density variables, using generalized linear mixed‐effects models (GLMMs) with binomial errors (Bolker et al. 2009). The GLMM is a logistic link function, in which the response variable is a logit‐transformed value of seedling fate: 1 (alive) or 0 (dead). The biotic and abiotic neighborhood variables were regarded as fixed effects. The random effects included quadrat and station levels to account for spatial autocorrelation in survival. Given the various ecological strategies, seedlings of diverse species were expected to respond differently to local neighborhood densities and habitat variables; we included species identity as a crossed random effect (Lin et al. 2012).

Seedling survival may vary among successional stages, size classes, and ages. In addition, we examined seedling height as a function of seedling age using a linear model. We found a significant positive relationship between seedling height and seedling age in three study areas (Table S4). Therefore, we included forest type as a fixed categorical effect and then examined three subsets of the data separately: (1) the community level (all species combined), (2) size classes (seedlings <20 cm in height and seedlings ≥20 cm in height), (3) different ages (1‐ to 2‐year‐old seedlings, 3‐ to 4‐year‐old seedlings, and seedlings 5 years or older). Within each subset, we tested the relative importance of different processes on seedling survival by comparing the following four models (Table 2): (1) a null model without fixed effect, (2) a biotic model containing seedling and adult neighborhood density variables as fixed effect, (3) an abiotic model containing habitat variables (canopy openness and soil nutrients) as fixed effect, and (4) a full model in which the biotic and abiotic variables are contained as fixed effect. The second model is compliant with the Janzen–Connell hypothesis only if conspecific neighborhoods have a stronger negative effect on the probability of seedling survival than heterospecific neighborhoods (Comita and Hubbell 2009). For each model, the goodness of fit was assessed based on Akaike's information criterion, and models with a difference between AIC values of <2 were considered as equivalent (AIC, Burnham and Anderson 2002). To access interspecific variation in the effect of the biotic neighborhood, we added a random effect with a species‐specific random slope to the coefficients of biotic neighborhood variable s in the best‐fitted model for each of the data subsets (Lin et al. 2012).

**Table 2:** Alternative models and their fixed effects

Model types	Fixed effects^a^
Null model	
Biotic model	FT + Cons + Hets + Cona + Heta
Abiotic model	FT + Soil PC1 + Soil PC2 + Soil PC3 + Soil PC4 + Soil PC5 + Canopy
Biotic + abiotic model	FT + Cons + Hets + Cona + Heta + Soil PC1 + Soil PC2 + Soil PC3 + Soil PC4 + Soil PC5 + Canopy

a Fixed effects include the following: FT (forest type), Cons (conspecific seedling density), Hets (heterospecific seedling density), Cona(conspecific adult tree density), Heta (heterospecific adult tree density), Soil PC1, Soil PC2, Soil PC3, Soil PC4, Soil PC5, and Canopy (canopy openness).

To investigate whether there was a CCT in our study area, we examined the probability of seedling survival against species population size (density or basal area of trees ≥1 cm DBH) using GLMMs.

All statistical analyses were implemented in the Program R 3.0.2 (R Development Core Team, 2013). GLMMs were fitted using the “lme4” package with a Laplace approximation method. The significance of fixed effects was assessed by Wald Z tests and the significance of random effects by likelihood ratio tests (Bolker et al. 2009). In the GLMMs, all continuous explanatory variables were standardized by subtracting the mean and dividing by the standard deviation before analysis. Odds ratios (OR) were also calculated for the estimate of each parameter. An OR >1 indicates positive effects on seedling survival, while OR < 1 indicates negative effects.

## Results

### Seedling composition and change in the three study areas

During the initial census in the summer and autumn (July–October) of 2012, altogether 1177 live seedlings (15 species) were counted in HF, 4110 (18 species) in MF, and 2216 (15 species) in OGF (Table S2). In these three study areas, 620 (53%), 2736 (67%), and 1764 (80%) seedlings did not survive during the following 12 months in the three areas, respectively. Survival rates varied among species and forest types (Table S5). The survival rates of individual species ranged from 7% to 100% (Table S5), while the mean survival rates by species were between 31% and 64% in the three study areas. The seedling survival rates were decreasing with increasing natural succession state in the study areas, in terms of both individual species and total seedling number (Table S5).

### The effect of biotic and abiotic neighborhoods at different size‐class levels

**Figure 2:**
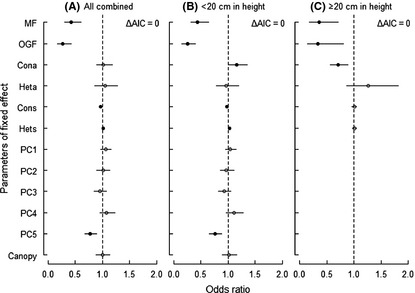
Odds ratios for model parameters estimated by the best models for all seedlings combined, seedlings <20 cm and seedlings ≥20 cm. Filled circles indicate significant effects. Horizontal lines indicate the 95% confidence intervals. MF and OGF means change in intercept relative to HF.

When analyzing all seedlings combined, the probability of seedling survival was best described by a full model, in which the effects of biotic and abiotic neighborhoods were combined (Table 3). Conspecific seedling neighborhoods and soil PC5 had significant negative effects on seedling survival (OR_Cons_ = 0.97, *P *<* *0.05; OR_PC5_ = 0.78, *P *<* *0.05; Fig. 2A), but no negative effect on conspecific adult neighborhoods (OR_Cona_ = 1.02, *P *>* *0.05; Fig. 2A). Heterospecific seedling neighborhoods, in turn, had a significant positive effect on seedling survival (OR_Hets_ = 1.02, *P *<* *0.05; Fig. 2A). Seedling survival rate in HF differed significantly with that in MF and OGF (OR_MF_ = 0.43, *P *<* *0.05; OR_OGF_ = 0.27, *P *<* *0.05; Fig. 2A), indicating that under the same level of biotic or abiotic factors, seedling survival decreased with succession advancement. For the smaller seedling cohort (<20 cm in height), the full model proved to be the best model (Table 3). The conspecific adult and heterospecific seedling neighborhoods showed a significant positive effect on seedling survival (OR_Cona_ = 1.16, *P *<* *0.05; OR_Hets_ = 1.02, *P *<* *0.05; Fig. 2B). Conspecific seedling neighborhoods and soil PC5 had significant negative effects on seedling survival (OR_Cons_ = 0.98, *P *<* *0.05; OR_PC5_ = 0.76, *P *<* *0.05; Fig. 2B). The difference on seedling survival between HF and other plots (MF and OGF) was significant (OR_MF_ = 0.44, *P *<* *0.05; OR_OGF_ = 0.25, *P *<* *0.05; Fig. 2B). For the larger seedling cohort (≥20 cm in height), the pattern of neighborhood effects for seedling survival was best described by the biotic model (Table 3). Conspecific adult neighborhoods, instead of conspecific seedling neighborhood, showed the only significant effect (OR_Cona_ = 0.71, *P *<* *0.05; Fig. 2C). Seedling survival rates were significantly different among successional stages (OR_MF_ = 0.36, *P *<* *0.05; OR_OGF_ = 0.34, *P *<* *0.05; Fig. 2C).

**Table 3:** Akaike's information criterion (AIC) and ΔAIC values for the generalized linear mixed models for survival

Data subsets	Model type							
	Null		Biotic		Abiotic		Biotic + Abiotic	
	AIC	ΔAIC	AIC	ΔAIC	AIC	ΔAIC	AIC	ΔAIC
All seedlings combined	8101.1	80.7	8024.2	3.8	8048.8	28.4	8020.4	**0.0**
Size cohort								
Seedlings <20 cm tall	7085.2	72.0	7019.3	6.1	7034.2	20.9	7013.2	**0.0**
Seedlings ≥20 cm tall	776.6	13.0	763.6	**0.0**	779.7	16.1	773.1	9.5
Age class								
1–2 year old	4560.3	37.8	4522.6	**0.0**	4531.1	8.5	4524.2	**1.6**
3–4 year old	2519.5	5.5	2515.1	**1.1**	2516.4	2.4	2514.0	**0.0**
≥5 year old	475.9	**0.4**	475.7	**0.3**	477.3	**1.9**	475.5	**0.0**

Bold values denote the best models based on the lowest AIC values.

### The effect of biotic and abiotic neighborhoods at different seedling ages

**Figure 3:**
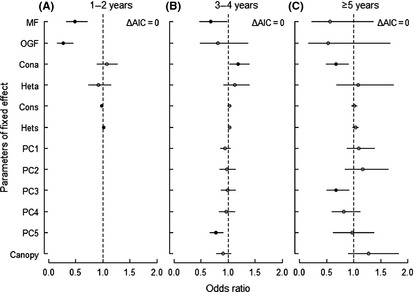
Odds ratios for model parameters estimated by the best models for seedlings of different age cohorts. Filled circles indicate significant effects. Horizontal lines indicate the 95% confidence intervals. MF and OGF means change in intercept relative to HF.

**Figure 4:**
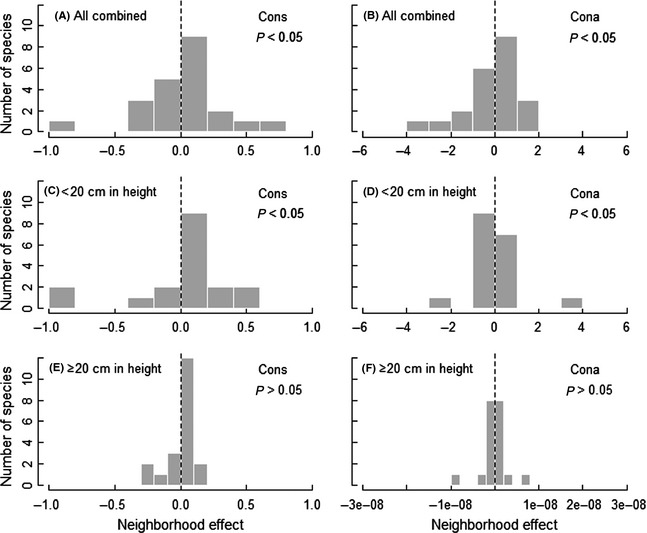
Distribution of the neighborhood effects on seedling survival and significance of the variation among species given the strength of the neighborhood effect under the likelihood ratio test for all seedlings combined (A, B), seedlings <20 cm in height (C, D) and seedlings ≥20 cm in height (E, F). This effect only includes that in the best‐fit models. The bars of the histograms are based on the coefficients of conspecific seedling and adult neighborhood variables for each species. Bars to the left of the dashed zero line indicate species whose survival is reduced by increasing neighborhood variables.

The neighborhood effect on seedling survival varied with seedling age (which was determined by counting annual bud scars, see methods section). The probability of survival for 1‐ to 2‐year‐old seedlings was best described by the biotic model and the full model (Table 3). There was a significantly negative effect of conspecific seedling neighborhoods and a significantly positive effect of heterospecific seedling neighborhoods (OR_Cons_ = 0.98, *P *<* *0.05; OR_Hets_ = 1.02, *P *<* *0.05; Fig. 3A). Seedling survival rates in MF and OGF were both significantly different compared with those in HF (OR_MF_ = 0.49, *P *<* *0.05; OR_OGF_ = 0.27, *P *<* *0.05; Fig. 3A). This is an indication that seedling survival decreases with the advancement of succession under the same level of biotic or abiotic factors. The effects on 3‐ to 4‐year‐old seedlings were best described by the full model as well as by the biotic model (Table 3), with a significant positive conspecific adult neighborhood effect and a significantly negative effect of the soil PC5 (OR_Cona_ = 1.18, *P *<* *0.05; OR_PC3_ = 0.77, *P *<* *0.05; Fig. 3B). Seedling survival rates in MF, in contrast to OGF, were significantly different from those in HF (OR_MF_ = 0.68, *P *<* *0.05; OR_OGF_ = 0.81, *P *>* *0.05; Fig. 3B). For seedlings older than 5 years, the seedling survival was best described by the full model with significantly negative effects from both conspecific neighboring adults and the soil PC3 (Table 3; OR_Cona_ = 0.67, *P *<* *0.05; OR _PC3_ = 0.67, *P *<* *0.05; Fig. 4C). Besides, the null model, the full and biotic models were the best models (Table 3). Except for the 1‐ to 2‐year old seedlings, the survival rate did not vary significantly among successional stages (OR_MF_ = 0.56, *P *>* *0.05; OR_OGF_ = 0.53, *P *>* *0.05; Fig. 3C).

### Variation in the strength of biotic neighborhood effects among species

To test the significance of the variation among species in the strength of neighborhood effects included in the best‐fit model, we compared models with and without variation among species regarding the effect of their biotic neighborhoods using a likelihood ratio test. For all seedlings combined and seedlings <20 cm in height, all the biotic neighborhood effects varied significantly among species, with positive relationship between survival and the biotic neighborhoods for some species and negative relationships for other species (Fig. 4A–D and Fig. S2A–D). For seedlings ≥20 cm in height, only the effect of heterospecific seedlings varied significantly across species, although here as well, the values approached zero with an overall slightly negative mean (Fig. 4E, F and Fig. S2E, F).

**Figure 5:**
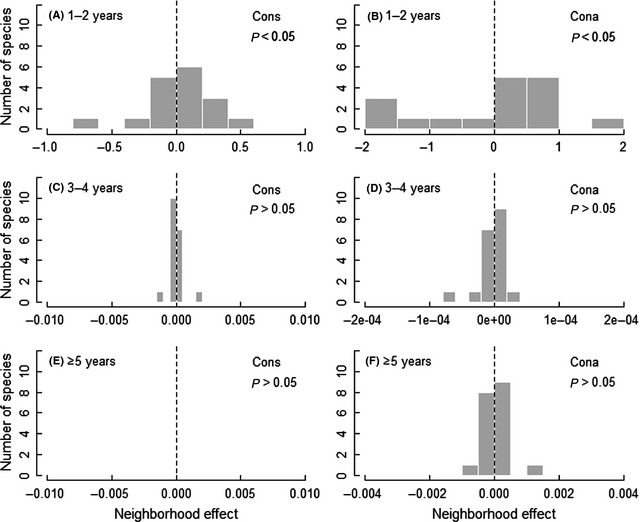
Distribution of the neighborhood effects on seedling survival and significance of the variation among species given the strength of the neighborhood effect under the likelihood ratio test for 1‐ to 2‐year‐old seedlings (A, B), 3‐ to 4‐year‐old seedlings (C, D) and seedlings ≥5 years old (E, F). This effect only includes that in the best‐fit models. The bars of the histograms are based on the coefficients of conspecific seedling and adult neighborhood variables for each species. Bars to the left of the dashed zero line indicate species whose survival is reduced by increasing neighborhood variables.

When separated into different age classes, we could only find significant variation in the strength of biotic neighborhood effects among species for 1‐ to 2‐year‐old seedlings (Fig. 5A, B and Fig. S3A, B). Neither the seedling nor the adult neighborhood effects varied significantly among species for seedlings older than 3 years (Fig. 5C–F and Fig. S3C–F).

### Community compensatory trend

**Figure 6:**
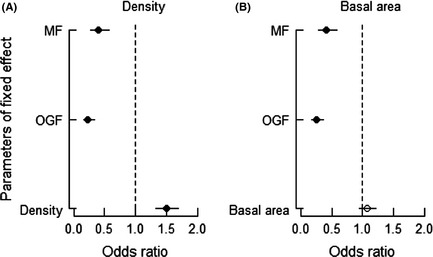
Odds ratios for model parameters estimated in GLMMs used to test for effects of population size (density or basal area) on seedling survival. MF and OGF mean change in intercept relative to HF.

We found a significant positive relationship between seedling survival and population density. The survival rates significantly varied among successional stages (OR_Density_ = 1.50, *P *<* *0.05; OR_MF_ = 0.40, *P *<* *0.05; OR_OGF_ = 0.23, *P *<* *0.05; Fig. 6A). However, the effect of basal area on survival was not significant, although there was significant variation among successional stages for seedling survival (OR_Basal area_ = 1.07, *P *<* *0.05; OR_MF_ = 0.41, *P *<* *0.05; OR_OGF_ = 0.25, *P *<* *0.05; Fig. 6B). This result suggests that a CCT, which is based on a negative relationship between seedling survival and population size, does not exist in any of the three forest types.

## Discussion

The results of our analysis present a convincing evidence of NDD, thus confirming previous studies (Packer and Clay 2000; Hille Ris Lambers and Clark 2003). These studies indicate that NDD has been widely accepted for temperate forests. The biotic and abiotic variables affecting seedling survival change with successional stage, seedling size, and age. Moreover, we found significant variation among species in the strength of their neighborhood effects for the smaller (<20 cm in height) and younger seedlings (1–2 years), as well as all seedlings combined. At the community level, inconsistent with CCT, we did not find a negative relationship between seedling survival, population density, or basal area. Our results confirm the importance of NDD and habitat niche partitioning in affecting differences in seedling survival between successional stage, seedling size, and age.

### Local neighborhood effect on seedling survival in different size classes

In this study, we found significant effects of both abiotic and biotic variables on seedling survival when analyzing all seedlings in the community together and seedlings <20 cm in height. The effects of the biotic neighborhood tended to be more important for seedlings ≥20 cm in height. This is an indication that the relative importance of abiotic and biotic variables on seedlings survival changes with seedling size. The smaller seedlings are more likely to be impacted by the biotic neighborhood and habitat factors than larger seedlings. One explanation is that seedlings of different sizes may differ in the susceptibility to biotic and abiotic stress (Bai et al. 2012). Some researchers have found that seedling height has a positive effect on the odds of survival, because taller plants may be less vulnerable to herbivores and pathogens than smaller plants (Queenborough et al. 2007; Comita et al. 2009; Chen et al. 2010). Otherwise, larger plants are better able to survive from the stresses of the understory, regardless of adult neighborhoods, providing them with the chance to take over the lead after persisting in the understory until a gap opens (Metz et al. 2010). In contrast, Lin et al. (2012) found a highly significant positive relationship between seedling height and their rate of survival in a tropical seasonal forest, but they did not find an essential difference when analyzing the data separately for seedlings of various sizes.

Furthermore, we found a significant positive relationship between seedling survival and heterospecific seedlings neighborhoods for seedlings <20 cm in height. One of the explanations for this result is the species herd protection hypothesis (Wills 1996; Peters 2003), which claims that increasing heterospecific neighborhood may facilitate focal seedling survival by depressing the probability of an encounter with a particular species‐specific pathogen. Another explanation refers to habitat preference. A species will tend to have a survival rate when growing in its preferred habitat (Wright 2002; Getzin et al. 2008), leading to a positive effect of conspecific neighborhoods on survival. However, Chen et al. (2010) who studied the seedling survival of 70 species in a subtropical forest found a significant negative correlation between heterospecific seedling density and relative conspecific seedling density using a permutation test. They insisted that if the relative density of conspecific seedlings is much lower than that of heterospecific seedlings, the effect of heterospecific seedling neighborhoods on seedling survival must be positive.

Besides, we found that NDD existed in both smaller and larger seedlings. The NDD for the smaller seedlings was caused by a conspecific seedling neighborhood and a conspecific adult neighborhood for the larger ones. It is likely that the enemy type (such as fungal pathogens, insects, and mammals) that results in NDD may vary during the life‐history stage (Fricke et al. 2014). Conversely, the effect of conspecific adult neighborhoods for the smaller seedlings was positive. This may probably be due to the fact that the smaller seedlings are aggregated around their parent trees as a result of dispersal limitation (He et al. 1997), thus generating a positive relationship between survival and conspecific adult neighborhoods.

### Local neighborhood effect on seedling survival at different ages

We also examined the neighborhood effect on seedling survival across ages and found that biotic neighborhoods have a strong effect on 1‐ to 2‐year‐old seedlings. This is consistent with the work of Bai et al. (2012) who found that the effect of biotic neighborhoods is more prevalent in young seedlings. For seedlings older than 3 years, survival was affected by both biotic and habitat factors. It is likely that, because 1‐ to 2‐year‐old seedlings are at the early stage of establishment and aggregate around the parent tree, they suffer strong intraspecific and interspecific competition for resources and are easy prey for natural enemies. Therefore, seedling survival tends to be more affected by biotic neighborhoods than by habitat factors. With increasing age, habitat plays an important role, consistent with the finding that habitat preferences contribute to explaining the abundance of woody species at the seedling stage (Norden et al. 2009). The effect of conspecific adult neighborhoods of seedlings older than 5 years became negative, suggesting that the effect of biotic factors on seedling survival manifested itself as NDD. This result agrees with Johnson et al. (2012), who analyzed the data of 151 species from more than 200,000 forest plots across a wide gradient and found that most species experienced conspecific negative density dependence.

In addition, we found evidence of NDD for 1‐ to 2–year‐old seedlings caused by conspecific seedling neighborhoods and for seedlings older than 5 years caused by conspecific adult neighborhoods. However, for seedlings of 3–4 years, although survival was affected by both biotic and abiotic variables, we did not find NDD. Conversely, the effect of conspecific adult neighborhoods was significantly positive for seedlings of 3–4 years. The newly recruited seedlings might have occurred in conspecific high density areas, which may result in density‐dependent mortality. Habitat preferences were an important factor that drives seedling survival (Comita et al. 2009).

### Variation among species in the strength of biotic neighborhood effect

We found wide variation among species in the strength of NDD on survival for the smaller (<20 cm in height, Fig. 4C and D) and the younger seedlings (1–2 years, Fig. 5A and B). This result suggests that the effects of NDD are not equivalent in all species, which is consistent with previous studies. For example, Comita et al. (2010) found that the effect of a conspecific neighborhood on seedling survival varied significantly among species in a tropical forest, while Lin et al. (2012) found wide variation among species in the strength of NDD over the dry‐season interval in a tropical rain forest. Nevertheless, we did not find significant variation among species in the strength of NDD or the larger (≥20 cm in height, Fig. 4E and F) and older seedlings (older than 3 years, Fig. 5C–F). This result indicates that the species‐specific variation in the strength of NDD may result from differences in seedlings resistance to biotic and abiotic stresses.

### Community compensatory trends

Most of the earlier studies provided conflicting evidence of the existence of CCTs in forests (Queenborough et al. 2007; Comita and Hubbell 2009; Chen et al. 2010; Metz et al. 2010; Bai et al. 2012). Although NDD affected seedling survival, surprisingly, we did not find a survival advantage for the less abundant species in our study areas. However, the rate of survival increased with an increasing density of conspecific trees. This observation is in contrast to previous results, where local‐scale density dependence could generate a community compensatory trend. Different ecological processes could thus lead to the same pattern. One explanation for this result is that the NDD and niche would tend to offset the CCT at the community level. On the other hand, previous studies on CCT were conducted over long periods (Queenborough et al. 2007), while we only used data over a survival period of 2 years. This may have limited our ability to detect a CCT.

## Conclusions

This study provides convincing evidence for the existence of NDD. Our results confirm that the biotic and abiotic variables affecting seedling survival change with successional stage, seedling size, and age. Moreover, we found significant variation among species in the strength of NDD for the smaller (<20 cm in height) and younger seedlings (1–2 years), as well as for all seedlings combined. This study also provides evidence that local‐scale density dependence will not always generate a community compensatory trend. The relative importance of NDD and habitat niche partitioning in driving seedling survival varies with seedling size and age in the study area, while the biotic and abiotic factors affecting seedlings survival change with successional stage.

## Conflict of Interest

None declared.

## Data Accessibility

Data ownership belongs to Beijing Forestry University, who conducted the analyses and wrote the manuscript http://www.bjfu.edu.cn/.

## Supporting information


**Table S1.** Basal area and density/ha by dominant tree species in the three forest plots.
**Table S2.** Number of seedlings for each species occurred in 2012 in the three forest plot.
**Table S3.** Soil variable loadings on the PCAs for the three forest plots.
**Table S4.** Coefficients and (standard errors) estimated in linear models for a relationship between seedling height and seedling age in three forest plot.
**Table S5.** Rates of seedling survival for each species in the HF, MF and OGF.
**Figure S1.** Census stations layout.
**Figure S2.** Distribution of the neighborhood effects on seedling survival and significance of the variation among species given the strength of the neighborhood effect under the likelihood ratio test for all seedlings combined (A, B), seedlings <20 cm in height (C, D) and seedlings ≥20 cm in height (E, F).
**Figure S3.** Distribution of the neighborhood effects on seedling survival and significance of the variation among species given the strength of the neighborhood effect under the likelihood ratio test for 1–2 year old seedlings (A, B), 3–4 year old seedlings (C, D) and seedlings ≥5 years old (E, F).
