# Supplementary material for: Drivers of seedling survival in a temperate forest and their relative importance at three stages of succession
Source: Ecol Evol. 2015 Sep 10;5(19):4287–99. doi: 10.1002/ece3.1688 (PMC4667830; doi:10.1002/ece3.1688)
Supplement: Supplementary file 1 — Table S1. Basal area and density/ha by dominant tree species in the three forest plots. Table S2. Number of seedlings for each species occurred in 2012 in the three forest plot. Table S3. Soil variable loadings on the PCAs for the three forest plots. Table S4. Coefficients and (standard errors) estimated in linear models for a relationship between seedling height and seedling age in three forest plot. Table S5. Rates of seedling survival for each species in the HF, MF and OGF. Figure S1. Census stations layout. Figure S2. Distribution of the neighborhood effects on seedling survival and significance of the variation among species given the strength of the neighborhood effect under the likelihood ratio test for all seedlings combined (A, B), seedlings <20 cm in height (C, D) and seedlings ≥20 cm in height (E, F). Figure S3. Distribution of the neighborhood effects on seedling survival and significance of the variation among species given the strength of the neighborhood effect under the likelihood ratio test for 1–2 year old seedlings (A, B), 3–4 year old seedlings (C, D) and seedlings ≥5 years old (E, F). [file ECE3-5-4287-s001.docx]

**Appendix**

**Table S1.** Basal area and density/ha by dominant tree species in the three forest plots

| Forest type | | Tree species | Total basal area (m2) | Stem density (ha) |
| --- | --- | --- | --- | --- |
| HF | 窗体顶端  *Juglans mandshurica* Maxim. 窗体底端 | 12.18 | 208.15 |  |
|  | 窗体顶端  *Fraxinus mandshurica* Rupr. 窗体底端 | 3.18 | 85.76 |  |
|  | 窗体顶端  *Ulmus davidiana Planch.* var. *japonica* (Rehder) Nakai 窗体底端 | 1.86 | 155.49 |  |
|  | 窗体顶端  *Acer mono* Maxim. 窗体底端 | 1.85 | 205.27 |  |
|  | 窗体顶端  *Pinus koraiensis* Siebold & Zucc. 窗体底端 | 1.83 | 29.30 |  |
| MF | 窗体顶端  *Acer mono* Maxim. 窗体底端 | 5.21 | 233.24 |  |
|  | 窗体顶端  *Pinus koraiensis* Siebold & Zucc*.* 窗体底端 | 4.28 | 61.12 |  |
|  | 窗体顶端  *Juglans mandshurica* Maxim. 窗体底端 | 3.66 | 50.40 |  |
|  | 窗体顶端  *Fraxinus mandshurica* Rupr. 窗体底端 | 3.59 | 52.19 |  |
|  | 窗体顶端  *Tilia amurensis* Rupr*.* 窗体底端 | 3.35 | 66.43 |  |
| OGF | 窗体顶端  *Ulmus laciniata* (Trautv.) Mayr. 窗体底端 | 4.44 | 92.40 |  |
|  | 窗体顶端  *Acer mono* Maxim. 窗体底端 | 4.02 | 134.20 |  |
|  | 窗体顶端  *Tilia amurensis* Rupr. 窗体底端 | 3.89 | 64.97 |  |
|  | 窗体顶端  *Pinus koraiensis* Siebold & Zucc. 窗体底端 | 3.05 | 43.83 |  |
|  | 窗体顶端  *Betula costata* Trautv. 窗体底端 | 2.46 | 27.40 |  |

**Table S2.** Number of seedlings for each species occurred in 2012 in the three forest plot

| Species code | Latin names | Family | Genus | Number of seedlings | | |
| --- | --- | --- | --- | --- | --- | --- |
|  |  |  |  | HF | MF | OGF |
| ACEMAN | *Acer mandshuricum* Maxim. | Aceraceae | Acer | 47 | 159 | 174 |
| PADRAC | *Padus racemosa* (Lam.) Gilib. | Rosaceae | Padus | 1 | - | - |
| ULMDAV | *Ulmus davidiana* *Planch.* var. *japonica* (Rehder) Nakai | Ulmaceae | Ulmus | 22 | 22 | 254 |
| ACEBAR | *Acer barbinerve* Maxim. | Aceraceae | Acer | 22 | 129 | 120 |
| ULMMAC | *Ulmus macrocarpa* Hance | Ulmaceae | Ulmus | - | 1 | - |
| PINKOR | *Pinus koraiensis* Siebold & Zucc. | Pinaceae | Pinus | 26 | 95 | 37 |
| JUGMAN | *Juglans mandshurica* Maxim | Juglandaceae | Juglans | 21 | 34 | 11 |
| PHEAMU | *Phellodendron amurense* Rupr. | Rutaceae | Phellodendron | - | 1 | 10 |
| TILMAN | *Tilia mandshurica* Rupr. & Maxim. | Tiliaceae | Tilia | - | 122 | 76 |
| ULMLAC | *Ulmus laciniata* (Trautv.) Mayr | Ulmaceae | Ulmus | 4 | 29 | 35 |
| CORMAN | *Corylus mandshurica* Maxim. | Betulaceae | Corylus | - | 3 | - |
| QUEMON | *Quercus mongolica* Fisch. ex Ledeb. | Fagaceae | Quercus | - | 11 | 10 |
| ACETRI | *Acer triflorum* Kom. | Aceraceae | Acer | - | 6 | - |
| CARCOR | *Carpinus cordata* Bl. | Betulaceae | Carpinus | 1 | 30 | 68 |
| ACETEG | *Acer tegmentosum* Maxim. | Aceraceae | Acer | 3 | 27 | 23 |
| POPCAT | *Populus cathayana* Rehder | Salicaceae | Populus | 23 | - | - |
| ACEMON | *Acer mono* Maxim. | Aceraceae | Acer | 108 | 539 | 298 |
| ABIHOL | *Abies holophylla* Maxim. | Pinaceae | Abies | 234 | 1392 | 1003 |
| POPDAV | *Populus davidiana* Dode | Salicaceae | Populus | 1 | - | - |
| RHADAV | *Rhamnus davurica* Pall. | Rhamnaceae | Rhamnus | 1 | - | - |
| FRAMAN | *Fraxinus mandshurica* Rupr. | Oleaceae | Fraxinus | 652 | 1429 | 71 |
| TILAMU | *Tilia amurensis* Rupr. | Tiliaceae | Tilia | 11 | 81 | 26 |
| Total |  |  |  | 1177 | 4110 | 2216 |

- Means the species was not existed in the plot.

**Table S3.** Soil variable loadings on the PCAs for the three forest plots

| Soil variables | HF | | | | |  | MF | | | | |  | OGF | | | | |
| --- | --- | --- | --- | --- | --- | --- | --- | --- | --- | --- | --- | --- | --- | --- | --- | --- | --- |
|  | PC1 | PC2 | PC3 | PC4 | PC5 |  | PC1 | PC2 | PC3 | PC4 | PC5 |  | PC1 | PC2 | PC3 | PC4 | PC5 |
| Total nitrogen | -0.39 | 0.44 | 0.13 | -0.43 | - |  | -0.32 | 0.30 | 0.51 | 0.25 | -0.37 |  | 0.56 | 0.21 | -0.19 | - | - |
| Total phosphorus | -0.25 | - | 0.56 | 0.47 | -0.38 |  | -0.44 | -0.26 | -0.24 | 0.28 | 0.32 |  | - | -0.60 | 0.13 | -0.17 | -0.45 |
| Total potassium | 0.37 | -0.25 | -0.33 | -0.36 | -0.34 |  | 0.37 | 0.44 | - | 0.32 | 0.42 |  | - | - | -0.81 | -0.56 | - |
| Organic matter | -0.43 | - | 0.10 | -0.58 | -0.26 |  | -0.38 | 0.52 | 0.22 | - | - |  | 0.61 | - | - | - | - |
| Available nitrogen | -0.47 | -0.26 | -0.18 |  | 0.38 |  | -0.42 | 0.31 | -0.37 | - | 0.39 |  | 0.51 | - | 0.14 | -0.11 | 0.17 |
| Available phosphorus | -0.15 | -0.71 | 0.24 | -0.11 | -0.32 |  | - | 0.24 | -0.69 | - | -0.63 |  | 0.14 | -0.42 | 0.34 | -0.53 | 0.44 |
| Available potassium | -0.40 | -0.29 | -0.42 | 0.24 | 0.17 |  | -0.48 | -0.38 | - | -0.17 |  |  | - | -0.41 | -0.37 | 0.45 | 0.65 |
| PH | -0.25 | 0.30 | -0.53 | 0.24 | -0.63 |  | - | 0.30 | - | -0.84 | 0.21 |  | 0.18 | -0.49 | -0.15 | 0.40 | -0.39 |
| Proportion of Variance | 0.33 | 0.16 | 0.15 | 0.13 | 0.08 |  | 0.30 | 0.17 | 0.14 | 0.13 | 0.10 |  | 0.27 | 0.17 | 0.13 | 0.12 | 0.11 |
| Cumulative Proportion | 0.33 | 0.50 | 0.65 | 0.78 | 0.86 |  | 0.30 | 0.47 | 0.61 | 0.75 | 0.85 |  | 0.27 | 0.44 | 0.57 | 0.69 | 0.80 |

- Means the term was not included in the PCA axis.

**Table S4.** Coefficients and (standard errors) estimated in linear models for a relationship between seedling height and seedling age in three forest plot

| Forest type | Intercept | age |
| --- | --- | --- |
| HF | -2.657(0.624) | 6.610(0.180) *** |
| MF | -1.556(0.231) | 5.823(0.074) *** |
| OGF | 0.197(0.380) | 5.296(0.128) *** |

**Table S5.** Rates of seedling survival for each species in the HF, MF and OGF.

| Species Code | Latin names | HF | MF | OGF |
| --- | --- | --- | --- | --- |
| ACEMAN | *Acer mandshuricum* Maxim. | 78.72 | 55.35 | 51.72 |
| PADRAC | *Padus racemosa* (Lam.) Gilib. | 100.00 | - | - |
| ULMDAV | *Ulmus davidiana Planch*. var. japonica (Rehder) Nakai | 63.64 | 45.45 | 25.98 |
| ACEBAR | *Acer barbinerve* Maxim. | 54.55 | 68.99 | 41.67 |
| ULMMAC | *Ulmus macrocarpa* Hance | - | 100.00 | - |
| PINKOR | *Pinus koraiensis* Siebold & Zucc. | 7.69 | 18.95 | 43.24 |
| JUGMAN | *Juglans mandshurica* Maxim | 52.38 | 20.59 | 27.27 |
| PHEAMU | *Phellodendron amurense* Rupr. | - | 0.00 | 20.00 |
| TILMAN | *Tilia mandshurica* Rupr. & Maxim. | - | 11.48 | 2.63 |
| ULMLAC | *Ulmus laciniata* (Trautv.) Mayr | 100.00 | 82.76 | 68.57 |
| CORMAN | *Corylus mandshurica* Maxim. | - | 0.00 | - |
| QUEMON | *Quercus mongolica* Fisch. ex Ledeb. | - | 27.27 | 10.00 |
| ACETRI | *Acer triflorum* Kom. | - | 50.00 | - |
| CARCOR | *Carpinus cordata* Bl. | 100.00 | 60.00 | 44.12 |
| ACETEG | *Acer tegmentosum* Maxim. | 66.67 | 59.26 | 43.48 |
| POPCAT | *Populus cathayana* Rehder | 60.87 | - | - |
| ACEMON | *Acer mono* Maxim. | 52.78 | 33.58 | 23.83 |
| ABIHOL | *Abies holophylla* Maxim. | 24.79 | 8.98 | 5.18 |
| POPDAV | *Populus davidiana* Dode | 100.00 | - | - |
| RHADAV | *Rhamnus davurica* Pall. | 100.00 | - | - |
| FRAMAN | *Fraxinus mandshurica* Rupr. | 52.30 | 53.95 | 46.48 |
| TILAMU | *Tilia amurensis* Rupr. | 9.09 | 7.41 | 7.69 |
| Total |  | 47.32 | 33.43 | 20.40 |
| Mean |  | 63.97 | 39.11 | 30.79 |

- Means the species was not existed in the plot.

**Figure S1.** Census stations layout. Solid circles indicate the focal seedling; hollow circles indicate conspecific seedlings; hollow triangles indicate heterospecific seedlings

**Figure S2.** Distribution of the neighborhood effects on seedling survival and significance of the variation among species given the strength of the neighborhood effect under the likelihood ratio test for all seedlings combined (a, b), seedlings < 20 cm in height (c, d) and seedlings ≥ 20 cm in height (e, f). This effect only includes that in the best-fit models. The bars of the histograms are based on the coefficients of heterospecific seedling and adult neighborhood variables for each species. Bars to the left of the dashed zero line indicate species whose survival is reduced by increasing neighborhood variables.

**Figure S3.** Distribution of the neighborhood effects on seedling survival and significance of the variation among species given the strength of the neighborhood effect under the likelihood ratio test for 1-2 year old seedlings (a, b), 3-4 year old seedlings (c, d) and seedlings ≥ 5 years old (e, f). This effect only includes that in the best-fit models. The bars of the histograms are based on the coefficients of heterospecific seedling and adult neighborhood variables for each species. Bars to the left of the dashed zero line indicate species whose survival is reduced by increasing neighborhood variables.
